# Predictive abilities of cardiovascular biomarkers to rapid decline of renal function in Chinese community-dwelling population: a 5-year prospective analysis

**DOI:** 10.1186/s12882-017-0743-y

**Published:** 2017-11-09

**Authors:** Shihui Fu, Chunling Liu, Leiming Luo, Ping Ye

**Affiliations:** 1 Department of Geriatric Cardiology, Chinese People’s Liberation Army General Hospital, Beijing, China; 2 Department of Cardiology and Hainan Branch, Chinese People’s Liberation Army General Hospital, Beijing, China; 3 Department of Nephrology and Hainan Branch, Chinese People’s Liberation Army General Hospital, Beijing, China

**Keywords:** Arterial stiffness and compliance, Cardiovascular biomarkers, Chinese community-dwelling population, Glomerular filtration rate, Rapid decline of renal function

## Abstract

**Background:**

Predictive abilities of cardiovascular biomarkers to renal function decline are more significant in Chinese community-dwelling population without glomerular filtration rate (GFR) below 60 ml/min/1.73m^2^, and long-term prospective study is an optimal choice to explore this problem. Aim of this analysis was to observe this problem during the follow-up of 5 years.

**Methods:**

In a large medical check-up program in Beijing, there were 948 participants with renal function evaluated at baseline and follow-up of 5 years. Physical examinations were performed by well-trained physicians. Blood samples were analyzed by qualified technicians in central laboratory.

**Results:**

Median rate of renal function decline was 1.46 (0.42–2.91) mL/min/1.73m^2^/year. Rapid decline of renal function had a prevalence of 23.5% (223 participants). Multivariate linear and Logistic regression analyses confirmed that age, sex, baseline GFR, homocysteine and N-terminal pro B-type natriuretic peptide (NT-proBNP) had independently predictive abilities to renal function decline rate and rapid decline of renal function (*p* < 0.05 for all). High-sensitivity cardiac troponin T (hs-cTnT), carotid femoral pulse wave velocity and central augmentation index had no statistically independent association with renal function decline rate and rapid decline of renal function (*p* > 0.05 for all).

**Conclusions:**

Homocysteine and NT-proBNP rather than hs-cTnT had independently predictive abilities to rapid decline of renal function in Chinese community-dwelling population without GFR below 60 ml/min/1.73m^2^. Baseline GFR was an independent factor predicting the rapid decline of renal function. Arterial stiffness and compliance had no independent effect on rapid decline of renal function. This analysis has a significant implication for public health, and changing the homocysteine and NT-proBNP levels might slow the rapid decline of renal function.

## Background

During the last few decades, rapid decline of renal function continues to grow in prevalence, and this trend is particularly obvious in patients with cardiovascular disease (CVD) [1]. Patients with CVD have clearly higher morbidity and mortality of chronic kidney disease (CKD) compared with those without CVD [2, 3]. As the established cardiovascular biomarkers, homocysteine, N-terminal pro B-type natriuretic peptide (NT-proBNP) and high-sensitivity cardiac troponin T (hs-cTnT) are considered to be elevated in patients with rapid decline of renal function, especially those with end stage renal disease (ESRD) [4, 5]. Previous studies have observed the relationships between cardiovascular biomarkers and renal function decline in patients with glomerular filtration rate (GFR) below 60 ml/min/1.73m^2^, and yielded the controversial results [6–8]. However, predictive abilities of cardiovascular biomarkers to renal function decline are more significant in community-dwelling population without GFR below 60 ml/min/1.73m^2^, and it is essential to analyze this problem in this population. Moreover, these cardiovascular biomarkers might play an etiologic role in renal function decline, and analyzing their relationships could promote the development of preventive strategies to slow the rapid decline of renal function.

Predictive abilities can not be fully evaluated by cross-sectional and short-term studies, and long-term prospective study is an optimal choice to explore the predictive abilities of cardiovascular biomarkers to renal function decline [9]. Moreover, this problem differs between racial groups, and there are few studies about this problem in China [10]. Therefore, aim of this prospective analysis was to observe the predictive abilities of cardiovascular biomarkers to renal function decline during the follow-up of 5 years in Chinese community-dwelling population without GFR below 60 ml/min/1.73m^2^.

## Methods

### Study population

This prospective analysis was performed in 1680 participants at least 18 years of age through a medical examination in Beijing, China. Participants were enrolled between May 2007 and July 2009, and the follow-up visit was conducted between February 2013 and September 2013. After 181 participants were lost, 1499 participants were followed up for 5 years. Exclusion criteria were death (52 participants), GFR below 60 ml/min/1.73m^2^ (12 participants) and missing values for variables (487 participants). The final study population was comprised of 948 participants.

### Physical examination

Physical examination was conducted by well-trained physicians. Resting blood pressure (BP) was determined by taking the mean of two measurements from the right arm of participants in the seated position with a standard mercury sphygmomanometer (Yuwell Medical Equipment & Supply Co., Ltd., Jiangsu, China). Hypertension was defined as mean systolic blood pressure (SBP) ≥ 140 mmHg, mean diastolic blood pressure (DBP) ≥ 90 mmHg, and/or use of any anti-hypertensive medications.

### Biochemical evaluation

Fasting blood samples were collected between 8 a.m. and 10 a.m., and sent to our central laboratory. Cardiovascular biomarkers were evaluated according to standard methods described by the manufacturers. Concentrations of fasting blood glucose (FBG), triglyceride (TG), high-density lipoprotein cholesterol (HDL-c) and low-density lipoprotein cholesterol (LDL-c) were quantified with Roche enzymatic assays (Roche Products Ltd., Basel, Switzerland) on a Roche autoanalyzer (Roche Products Ltd., Basel, Switzerland). Oral glucose tolerance test was done at timed intervals of two hours after drinking 75 g glucose load. Diabetes was defined as FBG ≥ 7.0 mmol/L, postprandial blood glucose (PBG) ≥ 11.1 mmol/L, and/or use of oral hypoglycemic medications or insulin. Concentrations of NT-proBNP were measured with electrochemiluminescence immunoassay (Roche Diagnostics GmbH) on an autoanalyzer (COBAS 6000; Roche Products Ltd., Basel, Switzerland). Concentrations of homocysteine were measured by high-performance chromatography with fluorometric detection. Concentrations of hs-cTnT were measured with Elecsys Troponin T high sensitive assay (Roche Products Ltd., Basel, Switzerland) by electrochemiluminescence immunoassay on a Modular Analytics E170 autoanalyzer (Roche Products Ltd., Basel, Switzerland). All assays were performed by qualified technicians without knowledge of clinical data.

### Arterial stiffness and compliance assessment

Central arterial stiffness was assessed by automated measurement of carotid femoral pulse wave velocity (cfPWV; Créatech, Besançon, France). cfPWV (m/s) was measured with two strain-gauge transducers (TY-306 Fukuda pressure-sensitive transducer; Fukuda Denshi Co., Tokyo, Japan) fixed transcutaneously over the carotid and femoral arteries (all on the right side), and then calculated from pulse transit time and distance between two recording sites [distance (m)/time (s)]. Central arterial compliance was assessed by automated measurement of central augmentation index (cAIx; SphygmoCor, Sydney, Australia). Central pressure waveform was obtained using its transfer function after mean radial artery waveform was calculated.

### Renal function decline

GFR was calculated using Chinese modified Modification of Diet in Renal Disease (MDRD) eq. [175 × plasma creatinine ^−1.234^ × age ^−0.179^ × 0.79 (if female)], where plasma creatinine was in mg/dL [11]. Concentrations of serum creatinine were measured with enzymatic assay (Roche Diagnostics GmbH) on a Hitachi 7600 autoanalyzer (Hitachi, Tokyo, Japan). Renal function decline rate was the change in GFR per year. Rapid decline of renal function was defined as a loss of GFR more than 3 ml/min/1.73m^2^ per year [12, 13].

### Statistical analysis

Continuous variables with normal distribution were described as mean (standard deviation) and compared with Student’s t-test. Continuous variables with skewed distribution were described as median (interquartile range) and compared with Mann–Whitney U test. Categorical variables were described as number (percentage) and compared with χ^2^ test. Baseline characteristics were compared between participants with and without rapid decline of renal function. Pearson and Spearman correlation analyses were used for simple correlation. Wilcoxon signed-rank test was conducted to compare the GFR levels at baseline and follow-up. Linear and Logistic regression analyses were conducted to observe the predictive abilities of cardiovascular biomarkers to renal function decline rate and rapid decline of renal function, respectively, after adjustment for age, sex, coronary artery disease, hypertension, diabetes, BMI, SBP, DBP, TG, HDL-c, LDL-c, FBG, PBG, baseline GFR, homocysteine, NT-proBNP, hs-cTnT, cfPWV and cAIx. Data were dealt with Statistical Package for Social Sciences (SPSS) version 17.0 (SPSS Inc., Chicago, IL, USA). Statistical significance referred to *p* < 0.05.

## Results

All participants had a median age of 60 (range: 25–96) years, and 44.6% (423 participants) were men (Table 1). Median baseline GFR level was 87.79 (79.27–96.89) mL/min/1.73m^2^, and median follow-up GFR level was 80.10 (72.02–87.89) mL/min/1.73m^2^ (*p* < 0.001 for change). Median rate of renal function decline was 1.46 (0.42–2.91) mL/min/1.73m^2^/year. Median levels of homocysteine and NT-proBNP were 16.75 (13.60–20.70) μmol/L and 38.41 (18.40–73.54) pg/ml, respectively. Rapid decline of renal function had a prevalence of 23.5% (223 participants). Age, baseline GFR, homocysteine, NT-proBNP and PBG levels, and proportions of men, hypertension and diabetes, were significantly higher in participants with rapid decline of renal function compared with those without rapid decline of renal function (*p* < 0.05 for all; Table 1).

**Table 1:** Characteristics of study population with and without rapid decline of renal function

Characteristics	Total population (*n* = 948)	With rapid decline of renal function (*n* = 223)	Without rapid decline of renal function (*n* = 725)	*P*-value
Age (year)	60(52–69)	61(53–71)	60(52–69)	0.033
Men (%)	423(44.6)	80(35.9)	343(47.3)	0.003
BMI (kg/m^2^)	25.32(23.37–27.76)	25.46(23.88–28.13)	25.27(23.19–27.64)	0.089
SBP (mmHg)	129(117–141)	130(119–140)	128(116–141)	0.701
DBP (mmHg)	77(70–84)	79(70–83)	77(70–84)	0.221
CAD (%)	107(11.3)	27(12.1)	80(11.0)	0.658
Hypertension (%)	471(49.7)	125(56.1)	346(47.7)	0.030
Diabetes (%)	246(25.9)	77(34.5)	169(23.3)	0.001
cfPWV (m/s)	10.7(9.3–12.6)	10.8(9.4–12.8)	10.6(9.3–12.6)	0.404
cAIx (%)	27(20–33)	28(22–33)	27(20–33)	0.090
TG (mmol/L)	1.47(1.08–2.11)	1.49(1.14–2.17)	1.47(1.07–2.10)	0.328
HDL-c (mmol/L)	1.34(1.13–1.55)	1.31(1.11–1.50)	1.35(1.14–1.59)	0.064
LDL-c (mmol/L)	2.89(2.42–3.29)	2.91(2.53–3.24)	2.88(2.40–3.31)	0.712
FBG (mmol/L)	4.97(4.52–5.48)	4.97(4.41–5.66)	4.97(4.54–5.46)	0.893
PBG (mmol/L)	6.54(5.28–8.46)	6.89(5.52–10.12)	6.40(5.26–8.25)	0.001
Homocysteine (μmol/L)	16.75(13.60–20.70)	17.30(14.70–20.90)	16.30(13.20–20.65)	0.006
NT-proBNP (pg/ml)	38.41(18.40–73.54)	41.55(22.50–84.77)	37.30(17.05–70.30)	0.017
Hs-cTnT (pg/ml)	4.00(3.00–8.19)	4.19(3.00–9.13)	4.00(3.00–7.92)	0.359
Baseline GFR (mL/min/1.73m^2^)	87.79(79.27–96.89)	102.01(89.94–113.83)	85.19(77.89–92.32)	<0.001
Follow-up GFR (mL/min/1.73m^2^)	80.10(72.02–87.89)	76.47(67.75–83.84)	81.19(73.27–88.85)	<0.001
△GFR/year (mL/min/1.73 m^2^/year)	1.46(0.42–2.91)	4.40(3.45–6.38)	1.01(0.08–1.81)	<0.001

Abbreviations: BMI: body mass index; cAIx: central augmentation index; CAD: coronary artery disease; cfPWV: carotid-femoral pulse wave velocity; DBP: diastolic blood pressure; FBG: fasting blood glucose; GFR: glomerular filtration rate. HDL-c: high-density lipoprotein cholesterol; hs-cTnT: high-sensitivity cardiac troponin T; LDL-c: low-density lipoprotein cholesterol; NT-proBNP: N-terminal pro B-type natriuretic peptide; PBG: postprandial blood glucose; SBP: systolic blood pressure; TG: triglyceride

Logistic regression analysis confirmed that not only age, sex and baseline GFR levels, but also homocysteine and NT-proBNP levels had independently predictive abilities to renal function decline rate (NT-proBNP: *p* = 0.001; others: *p* < 0.001 for all; Table 2). Levels of hs-cTnT, cfPWV and cAIx had no statistically independent association with renal function decline rate (*p* > 0.05 for all). Linear regression analysis confirmed that not only age, sex, baseline GFR levels and hypertension, but also homocysteine and NT-proBNP levels had independently predictive abilities to rapid decline of renal function (hypertension: 0.029; NT-proBNP: *p* = 0.027; others: *p* < 0.001 for all; Table 3). Levels of hs-cTnT, cfPWV and cAIx had no statistically independent association with rapid decline of renal function (*p* > 0.05 for all).

**Table 2:** Predictive abilities of cardiovascular biomarkers to renal function decline rate in simple correlation and linear regression analyses

Characteristics	r	*P*-value	β	*P*-value
Age (year)	0.057	0.078	0.180	<0.001
Men (%)	0.124	<0.001	0.142	<0.001
BMI (kg/m^2^)	0.049	0.132	0.020	0.481
SBP (mmHg)	0.015	0.640	−0.026	0.517
DBP (mmHg)	−0.001	0.973	0.009	0.799
CAD (%)	0.060	0.067	0.017	0.526
Hypertension (%)	0.057	0.077	0.062	0.070
Diabetes (%)	0.128	<0.001	−0.008	0.821
cfPWV (m/s)	−0.002	0.939	−0.023	0.476
cAIx (%)	0.071	0.029	<0.001	0.998
TG (mmol/L)	0.040	0.224	−0.033	0.244
HDL-c (mmol/L)	−0.038	0.238	−0.038	0.211
LDL-c (mmol/L)	0.045	0.165	0.016	0.554
FBG (mmol/L)	−0.037	0.251	0.028	0.503
PBG (mmol/L)	0.109	0.001	0.052	0.271
Homocysteine (μmol/L)	0.116	<0.001	0.158	<0.001
NT-proBNP (pg/ml)	0.081	0.013	0.088	0.001
Hs-cTnT (pg/ml)	0.044	0.179	0.014	0.599
Baseline GFR (mL/min/1.73m^2^)	0.480	<0.001	0.620	<0.001

Abbreviations: BMI: body mass index; cAIx: central augmentation index; CAD: coronary artery disease; cfPWV: carotid-femoral pulse wave velocity; DBP: diastolic blood pressure; FBG: fasting blood glucose; GFR: glomerular filtration rate; HDL-c: high-density lipoprotein cholesterol; hs-cTnT: high-sensitivity cardiac troponin T; LDL-c: low-density lipoprotein cholesterol; NT-proBNP: N-terminal pro B-type natriuretic peptide; PBG: postprandial blood glucose; SBP: systolic blood pressure; TG: triglyceride

**Table 3:** Predictive abilities of cardiovascular biomarkers to rapid decline of renal function in Logistic regression analysis

Characteristics	HR	95CI	*P*-value
Age (year)	1.057	1.032–1.084	<0.001
Men (%)	2.531	1.564–4.096	<0.001
BMI (kg/m2)	1.021	0.962–1.083	0.493
SBP (mmHg)	0.987	0.971–1.003	0.100
DBP (mmHg)	1.019	0.994–1.045	0.132
CAD (%)	0.681	0.365–1.269	0.227
Hypertension (%)	1.740	1.059–2.857	0.029
Diabetes (%)	0.743	0.436–1.266	0.275
cfPWV (m/s)	0.985	0.905–1.072	0.726
cAIx (%)	0.994	0.974–1.015	0.561
TG (mmol/L)	0.900	0.744–1.089	0.280
HDL-c (mmol/L)	0.622	0.330–1.171	0.141
LDL-c (mmol/L)	0.919	0.693–1.219	0.558
FBG (mmol/L)	1.121	0.935–1.343	0.217
PBG (mmol/L)	1.061	0.976–1.153	0.162
Homocysteine (μmol/L)	1.048	1.024–1.072	<0.001
NT-proBNP (pg/ml)	1.001	1.000–1.003	0.027
Hs-cTnT (pg/ml)	1.011	0.993–1.030	0.220
Baseline GFR (mL/min/1.73 m^2^)	1.117	1.098–1.136	<0.001

Abbreviations: BMI: body mass index; cAIx: central augmentation index; CAD: coronary artery disease; cfPWV: carotid-femoral pulse wave velocity; CI: confidence interval; DBP: diastolic blood pressure; FBG: fasting blood glucose; GFR: glomerular filtration rate; HDL-c: high-density lipoprotein cholesterol; hs-cTnT: high-sensitivity cardiac troponin T; LDL-c: low-density lipoprotein cholesterol; NT-proBNP: N-terminal pro B-type natriuretic peptide; PBG: postprandial blood glucose; HR: hazard ratio; SBP: systolic blood pressure; TG: triglyceride

## Discussion

Predictive abilities of cardiovascular biomarkers to rapid decline of renal function have been mainly discussed in clinical patients, and few studies have been performed in community-dwelling population without GFR below 60 ml/min/1.73m^2^ [6–8]. Moreover, most of conflicting evidences have been provided by cross-sectional and short-term studies, and it is essential to perform the long-term prospective study to analyze this problem [9]. Almost no long-term prospective study has evaluated this problem in China [10]. This prospective analysis had the following findings during the follow-up of 5 years in Chinese community-dwelling population without GFR below 60 ml/min/1.73m^2^: 1. homocysteine and NT-proBNP rather than hs-cTnT had the independent abilities to predict the rapid decline of renal function, with their higher levels indicating more rapid renal function decline rate; 2. baseline GFR was an independent factor predicting the rapid decline of renal function; 3. elderly and females had a more rapid decline of renal function compared with others; 4. role of hypertension in rapid decline of renal function could not be ignored; 5. arterial stiffness and compliance had no independent effect on rapid decline of renal function.

Cross-sectional researches have realized that homocysteine levels were negatively related to GFR in black and white adults [14]. However, an increase in homocysteine levels was not an independent risk factor for renal disease in a study with the follow-up of 2.2 years [15]. During the follow-up of 5 years, this prospective analysis demonstrated the independently predictive ability of homocysteine to not only renal function decline rate, but also rapid decline of renal function, strongly supporting the adverse effect of homocysteine on renal function. Homocysteine might play an etiologic role in renal function decline through injuring the renal blood vessels. As a pro-oxidant, homocysteine could diminish the nitric oxide-mediated vasodilation, promote the thrombosis and impede the fibrinolysis [16–19]. Elevated homocysteine levels could be a target of future intervention studies to slow the rapid decline of renal function, and folic acid might be a choice in clinical therapy of renal injury.

Professor Seki N has proposed that natriuretic peptide was an independent risk factor for renal function decline rate [20]. Professor Spanaus KS has reported that elevated NT-proBNP levels indicated an increased risk for accelerated progression of renal disease [21]. This prospective analysis, directing at Chinese community-dwelling populationn without GFR below 60 ml/min/1.73m^2^, identified the NT-proBNP as an independent risk factor for rapid decline of renal function. Endogenous NT-proBNP at physiological levels affects the glomerular filtration and renal function [22]. Glomerular hyperfiltration induces the glomerular hypertension and stretches the mesangial cells. Stretched cells secrete the cytokines that stimulate the production of extracellular matrix proteins, accumulation of which promotes the progression of renal injury. Moreover, natriuretic peptide receptor antagonist or angiotensin receptor blockade and neutral endopeptidase inhibition (ARNI, LCZ696) might be useful to prevent the renal injury [23, 24]. Therefore, effective monitoring of NT-proBNP levels could slow the rapid decline of renal function.

Age and baseline GFR have been suggested to correlate with ESKD [25]. Based on Epidemiologia do Idoso (EPIDOSO) Study, age and baseline GFR were associated with progressive decline in renal function [26]. Professor Seki N has realized that baseline GFR had significantly positive association with renal function decline rate [27]. This prospective analysis discovered in Chinese community-dwelling population without GFR below 60 ml/min/1.73m^2^ that baseline GFR was significantly related to rapid decline of renal function, which was consistent with prior finding that glomerular hyperfiltration was a determinant of renal function decline [28, 29]. Accordingly, the correction of glomerular hyperfiltration might be valuable for slowing the rapid decline of renal function. Meanwhile, the finding from Cardiovascular Health Study has suggested that elevated BP contributed to renal function decline in elderly [30]. According to Tromso Study, high BP predicted a decline in GFR [31]. However, Leiden 85-Plus Study has found that low BP was related to renal function decline in elderly [32]. This prospective analysis demonstrated a potential effect of hypertension on rapid decline of renal function in Chinese community-dwelling population without GFR below 60 ml/min/1.73m^2^.

Professor Ford ML has suggested that arterial stiffness was related to renal function decline rate [33]. In 482 community-dwelling individuals free from ESRD, renal function decline was associated with increased arterial stiffness [34]. However, professor Kim CS has illustrated that arterial stiffness was not associated with rapid decline of renal function in participants without GFR < 30 mL/min/1.73m^2^ [35]. In this prospective analysis, arterial stiffness and compliance had no statistically independent effect on rapid decline of renal function in Chinese community-dwelling population without GFR below 60 ml/min/1.73m^2^.

The findings of this prospective analysis had public health relevance. Rapid decline of renal function has a growing prevalence in China. Given that there are limited interventions available, public health initiatives are needed for slowing the rapid decline of renal function. This prospective analysis confirmed that elevated homocysteine and NT-proBNP levels contributed to rapid decline of renal function. Future studies are required to determine whether therapies changing the homocysteine and natriuretic peptide levels ultimately affect the rapid decline of renal function. Effects of folic acid, natriuretic peptide receptor antagonist, angiotensin receptor blockade and neutral endopeptidase inhibition (LCZ696) and other medications associated with homocysteine and NT-proBNP levels on renal function are necessary to be paid special attention in pharmaceutical research and clinical practice.

The current analysis had some limitations. Firstly, 487 participants (30%) were excluded due to missing values for variables, and it was difficult to determine their all variable information. However, there were only 181 participants lost during the follow-up of 5 years. Secondly, MDRD equation rather than Chronic Kidney Disease Epidemiology Collaboration (CKD-EPI) equation was applied to evaluate renal function in the current analysis. However, MDRD equation is more commonly applied in epidemiological investigation compared with CKD-EPI equation. Moreover, MDRD equation but not CKD-EPI equation has Chinese modified version (CMDRD). CMDRD equation is more suitable for Chinese community-dwelling population and has superior accuracy than CKD-EPI equation.

## Conclusions

This prospective analysis demonstrated that homocysteine and NT-proBNP rather than hs-cTnT had independently predictive abilities to rapid decline of renal function in Chinese community-dwelling population without GFR below 60 ml/min/1.73m^2^. Moreover. baseline GFR was an independent factor predicting the rapid decline of renal function. Meanwhile, arterial stiffness and compliance had no independent effect on rapid decline of renal function. This prospective analysis has a significant implication for public health, and further studies are warranted to establish the benefit of interventions changing the homocysteine and NT-proBNP levels on slowing the rapid decline of renal function.

## Abbreviations

BMI: body mass index
cAIx: central augmentation index
cfPWV: carotid femoral pulse wave velocity
DBP: diastolic blood pressure
FBG: fasting blood glucose
GFR: glomerular filtration rate
HDL-c: high-density lipoprotein cholesterol
hs-cTnT: high-sensitivity cardiac troponin T
LDL-c: low-density lipoprotein cholesterol
NT-proBNP: N-terminal pro B-type natriuretic peptide
PBG: postprandial blood glucose
SBP: systolic blood pressure
TG: triglyceride
